# Irish Medical Corporations

**Published:** 1836-04

**Authors:** 


### IRISH MEDICAL CORPORATIONS.

No. 1. *Medical Degrees* (M.B., M.D.,) *granted by the Trinity College, Dublin, during the last Ten Years.*

| Years ....... .. | 1826 | 1827 | 1828 | 1829 | 1830 | 1831 | 1832 | 1833 | 1834 | 1835 | Total |
|---|---|---|---|---|---|---|---|---|---|---|---|
| Degrees ... .. | 4 | 18 | 16 | 10 | 10 | 17 | 19 | 16 | 19 | 16 | 145 |

*Note.*—The proportion of M.B. to M.D. degrees may be reckoned, perhaps, as three to one. Very few take the degree of M.D., except such as intend practising in London, and who take an *ad eundem* degree at one of the English universities. There was no "commencement" in 1826, which accounts for the small number of graduates.

No. 2. *Admissions into the King and Queen's College of Physicians, in Ireland, during Ten Years.*

| | 1826 | 1827 | 1828 | 1829 | 1830 | 1831 | 1832 | 1833 | 1834 | 1835 | Total |
|---|---|---|---|---|---|---|---|---|---|---|---|
| Fellows - | 2 | 4 | 2 | 8 | 3 | 1 | 4 | 2 | 0 | 2 | 28 |
| Licentiates | 9 | 5 | 8 | 7 | 1 | 5 | 5 | 2 | 0 | 1 | 43 |
| Total | 11 | 9 | 10 | 15 | 4 | 6 | 9 | 4 | 0 | 3 | 71 |

No. 3. *Diplomas granted by the College of Surgeons in Ireland during Ten Years.*

| Years ...................... | 1826 | 1827 | 1828 | 1829 | 1830 | 1831 | 1832 | 1833 | 1834 | 1835 | Total |
|---|---|---|---|---|---|---|---|---|---|---|---|
| Number of Licentiates... | 14 | 23 | 35 | 29 | 37 | 37 | 27 | 39 | 44 | 29 | 314 |

No. 4. *Licences granted by Apothecaries' Hall in Ireland, during the last Ten Years.*

| Years ......... | 1826 | 1827 | 1828 | 1829 | 1830 | 1831 | 1832 | 1833 | 1834 | 1835 | Total |
|---|---|---|---|---|---|---|---|---|---|---|---|
| Licentiates ... | 44 | 87 | 92 | 66 | 52 | 33 | 34 | 55 | 50 | 59 | 572 |

